# Overcoming the problems caused by collinearity in mixed-effects logistic model: determining the contribution of various types of violence on depression in pregnant women

**DOI:** 10.1186/s12874-021-01325-7

**Published:** 2021-07-28

**Authors:** Sanaz Khalili, Javad Faradmal, Hossein Mahjub, Babak Moeini, Khadijeh Ezzati-Rastegar

**Affiliations:** 1 Department of Biostatistics School of Public Health, Hamadan University of Medical Sciences, Hamadan, Iran; 2 Department of Biostatistics School of Public Health, Modeling of Noncommunicable Diseases Research Center, Hamadan University of Medical Sciences, Hamadan, Iran; 3 Social Determinants of Health Research Center, Hamadan University of Medical Sciences, Hamadan, Iran; 4 Health Education and Promotion, Department of Public Health, Hamadan University of Medical Sciences, Hamadan, Iran

**Keywords:** Collinearity, Mixed-effects logistic model, Ridge estimator, Violence

## Abstract

**Background:**

Collinearity is a common and problematic phenomenon in studies on public health. It leads to inflation in variance of estimator and reduces test power. This phenomenon can occur in any model. In this study, a new ridge mixed-effects logistic model (RMELM) is proposed to overcome consequences of collinearity in correlated binary responses.

**Methods:**

Parameters were estimated through penalized log-likelihood with combining expectation maximization (EM) algorithm, gradient ascent, and Fisher-scoring methods. A simulation study was performed to compare new model with mixed-effects logistic model(MELM). Mean square error, relative bias, empirical power, and variance of random effects were used to evaluate RMELM. Also, contribution of various types of violence, and intervention on depression among pregnant women experiencing intimate partner violence(IPV) were analyzed by new and previous models.

**Results:**

Simulation study showed that mean square errors of fixed effects were decreased for RMELM than MELM and empirical power were increased. Inflation in variance of estimators due to collinearity was clearly shown in the MELM in data on IPV and RMELM adjusted the variances.

**Conclusions:**

According to simulation results and analyzing IPV data, this new estimator is appropriate to deal with collinearity problems in the modelling of correlated binary responses.

## Introduction

Intimate partner violence (IPV) against women is one of the major public health challenges in the world [1]. IPV is categorized into mental, physical, sexual, and financial types [2]. IPV can cause physical problems including bruising, fractures, trauma, and various sexually transmitted infections. It can also cause mental health problems in women, such as depression, anxiety, and even suicide [3]. There are many women who may experience depression during pregnancy and the risk of it increases under IPV [4, 5]. Some interventions may be useful to reduce odds of depression in pregnant women under IPV. For analyzing these longitudinal studies with binary responses, mixed-effects logistic model (MELM) is used as a common model. Usually, this method estimates the fixed parameters based on maximum likelihood and uses the adjusted Gauss-Hermite to approximate the integral related to random effects [6, 7]. Modeling of the correlated binary responses may suffer from some problems in modeling like collinearity [8].

Collinearity is referred to the linear relationship between predictor variables. The inherent relationship between variables in the real world, small sample size, design of model, and the trend of predictor variables can cause collinearity [9]. Indeed, the issue that makes collinearity an important problem in modeling is variance of estimators. When there is collinearity, determinant of *X*^*T*^*X* becomes small, where *X* is design matrix, leading to an inflation in variance of estimators. Bias in decision on predictor variables and wide confidence interval length are other consequences of collinearity. In addition, collinearity makes the effects of predictor variables inseparable and it may be difficult to evaluate relative importance of each predictor variable [8, 10, 11].

There are some simple methods to deal with collinearity. Drawing back collinear variables, centering predictor variables, and using dimension reduction methods like principal component analysis are some of these solutions. But, it should be mentioned that despite simplicity, each of which has their own disadvantages [12, 13]. Ridge estimator is one of the methods shown a desirable effect against consequences of collinearity. In this method, the penalized log-likelihood is used with ridge penalty. Then, ridge estimator imposes some bias to estimator, by adding a constant value in the main diagonal of *X*^*T*^*X* but decreases its variance. In fact, it is a tradeoff between bias and variance [11, 12, 14, 15].

Various studies have been conducted to compare performance of ridge, lasso, and Firth penalties. For instance, studies have shown that problems can arise if lasso penalty is applied instead of ridge penalty in the presence of collinearity. The first problem is variable selection. In the presence of collinearity between variables, lasso method randomly removes one variable from the model. The second problem is prediction accuracy of the model. Prediction accuracy of lasso method is less than ridge[16] and mean square error of ridge method is less than lasso method [17, 18]. Also, in the presence of separation, the use of Firth penalty compared to ridge leads to more accurate estimates [19].

MELM uses maximum likelihood estimator (MLE) for estimation of the fixed effects. So, inflation in variance of the estimator and lack of significance in important variables may occur. Due to the increase in the number of studies with the correlated binary responses, such as longitudinal and cluster studies, in this paper, a ridge estimator is proposed in the correlated binary responses based on Fahrmeir and Tutz method [20, 21]. Herein, the details on method and estimators are introduced in Method section. Analysis of IPV data and simulation study are presented in Numerical study section. Finally, discussion of findings and conclusions are provided in Discussion and conclusions section.

## Method

Suppose *y*_*ij*_ determines the *j*th observation for *i*th individual, *i*=1,2,...,*n*, and *j*=1,2,...,*n*_*i*_. MELM is defined as:

1 $$ log\left(\frac{{\pi}_{ij}}{1-{{\pi}_{ij}}}\right) = \mathbf{x}_{ij}^{T} \pmb{\beta} + {\mathbf{z}^{T}_{ij}} \mathbf{b}_{i},  $$

where, **x**_*ij*_ and **z**_*ij*_ are observation vector for fixed and random effect for *i*th individual in *j* observation, respectively. *X* and *Z* are the design matrix for the fixed and random effect. Vector of the fixed and random effect are denoted by *β*_*p*×1_ and **b**_*i*_. Penalized log-likelihood with Breslow and Clayton integral approximation for model 1 is in the form of 2.

2 $$ \begin{aligned} l^{\lambda}=& \sum_{i=1}^{n} \sum_{j=1}^{n_{i}}\left(y_{ij}\left(\mathbf{x}_{ij}^{T} \pmb{\beta} + {\mathbf{z}_{ij}}^{T} \mathbf{b}_{i}\right)\right)-log\left(1 + exp\left(\mathbf{x}_{ij}^{T} \pmb{\beta} + \mathbf{z}_{ij}^{T} \mathbf{b}_{i}\right)\right)\\ &- \lambda ~\pmb{\beta}^{T} \pmb{\beta} - \frac{1}{2} \mathbf{b}^{T} Q^{-1} \mathbf{b}, \end{aligned}  $$

where *Q* is covariance matrix for distribution of random effects and **b**^*T*^=(**b**_1_,**b**_2_,...,**b**_*n*_). The ridge shrinkage parameter is shown as *λ*. The fixed and random effects vectors are considered in the form of one vector, and all subsequent calculations are performed based on this new vector. This vector is defined as *δ*^*T*^=(*β*^*T*^,*b*^*T*^) where, $\hat {\pmb {\delta }}$ is a vector maximizing 2. Let *A*=[*X*,*Z*],*U*=*diag*(0,...,0,*Q*^−1^,...,*Q*^−1^) such that, *U* is a block-diagonal matrix with *p* zeros and *n* times the inverse of covariance matrix. Then, the Fisher information matrix is calculated, $F^{\lambda }(\hat {\pmb {\delta }})$, as ${{F}^{\lambda }}(\hat {\pmb {\delta }})=A^{T} \hat {\Psi } (\hat {\pmb {\delta }}) A +U + \lambda ^{(s-1)};$ where $ \hat {\Psi }(\hat {\pmb {\delta }}) = D (\hat {\pmb {\delta }}) ~ \nu ^{-1}(\hat {\pmb {\delta }})~ D^{T}(\hat {\pmb {\delta }}), D (\hat {\pmb {\delta }}) =\frac {\partial h(\pmb {\eta })}{\partial {\pmb {\eta }}}$ and $\nu (\hat {\pmb {\delta }})=cov(y|\pmb \delta)$. Here, $\eta _{ij}= \mathbf {x}_{ij}^{T} \pmb {\beta } + {\mathbf {z}^{T}_{ij}} \mathbf {b}_{i} $, and $\phantom {\dot {i}\!}{\pmb {\eta }}^{T}=(\eta _{11},..., \eta _{1n_{1}},..., \eta _{n1},..., \eta _{nn_{n}})$. The form of Fisher information matrix is as:

$${{{F}^{\lambda}}}(\hat{\pmb {\delta}})= \left[ \begin{array}{ccccc} F^{\lambda}_{{\pmb \beta \pmb\beta}} & F^{\lambda}_{{\pmb \beta 1}} & F^{\lambda}_{{\pmb \beta 2}} &...& F^{\lambda}_{{\pmb \beta n}} \\ F^{\lambda}_{1 {\pmb \beta }} & F^{\lambda}_{11}&&&0\\ F^{\lambda}_{2 {\pmb \beta }}&& F^{\lambda}_{22}\\ \vdots&&&\ddots&\\ F^{\lambda}_{n \pmb \beta}&0&&&F^{\lambda}_{nn} \end{array} \right] $$

For performing optimization, derivative is taken from the penalized log-likelihood and it is represented by **s**^*λ*^(*δ*):

3 $$ \mathbf{s}^{\lambda}(\pmb \delta) =\left\{ \begin{array}{l} \frac{\partial l^{\lambda}}{\partial \pmb {\beta}}= \sum_{i=1}^{n} \sum_{j=1}^{n_{i}}(y_{ij} \mathbf{x}_{ij} - \pi_{ij} \mathbf{x}_{ij})- 2\lambda \pmb \beta \\ \\ \frac{\partial l^{\lambda}}{\partial \mathbf{b_{i}}}= \sum_{i=1}^{n} \sum_{j=1}^{n_{i}}(y_{ij} \mathbf{z}_{ij} - \pi_{ij} \mathbf{z}_{ij})- 2Q^{-1} \mathbf{b}_{i} \end{array}. \right.  $$

Here, two optimization methods are combined to increase convergence speed. For estimating *δ*, the gradient ascent and Fisher-scoring methods are used:

$$\hat{\pmb \delta}^{s} = \left\{ \begin{array}{l} \hat{\pmb \delta}^{s - 1} +{\vartheta}^{s - 1} ~\mathbf{s}^{\lambda}\left(\hat{\pmb \delta}^{s - 1}\right)\\ \\ \hat{\pmb \delta}^{s - 1} + \left({F^{\lambda}}\left(\hat{\pmb \delta}^{s - 1}\right) \right)^{- 1} ~\mathbf{s}^{\lambda}\left(\hat{\pmb \delta}^{s - 1}\right) \end{array}. \right. $$

where *𝜗* is step size:

$$\vartheta=\frac{\left(\mathbf{s}^{\lambda}\left(\hat{\pmb \delta}\right)\right)^{T} ~~\mathbf{s}^{\lambda}(\hat{\pmb \delta}) } {\left(\mathbf{s}^{\lambda}\left(\hat{\pmb \delta}\right)\right)^{T} ~~F^{\lambda}(\hat{\pmb \delta}) ~~\mathbf{s}^{\lambda}(\hat{\pmb \delta}) }~. $$

To estimate the variance component, the EM algorithm is used. The estimation of variance is:

4 $$  \hat{Q}^{(s)} = \frac{1}{n}\sum_{i=1}^{n} \left(\hat{\mathbf{v}}_{ii}^{(s)} + \hat{\mathbf{b}}_{i}^{(s)} \left(\hat{\mathbf{b}}_{i}^{(s)} \right)^{T}\right),  $$

where $\mathbf {v}_{ii}= {F^{\lambda }}_{ii}^{-1} +~ {F^{\lambda }}_{ii}^{-1} ~{F^{\lambda }}_{i \pmb \beta }\left ({F^{\lambda }}_{\pmb \beta \pmb \beta } - \sum _{i=1}^{n}{F^{\lambda }}_{\pmb \beta i} ~ { F^{\lambda }}^{-1}_{ii} {F^{\lambda }}_{i \pmb \beta }{\vphantom {{F^{\lambda }}_{ii}^{-1}}}\right)^{-1} {F^{\lambda }}_{i \pmb \beta } ~{F^{\lambda }}_{ii}^{-1}. $

### Shrinkage parameter

The shrinkage parameter was obtained through $ \lambda =\prod \limits _{k=1}^{p} \left (\frac {1}{m_{k}}\right)^{\frac {1}{p}}$, where *p* is the number of predictor variables. Here, ${m_{k}}=\sqrt {\frac {{\hat {\sigma }}^{2}}{{{\hat {\alpha }_{k}}}^{2}}}$, and $\hat {\alpha }_{k}$ is the *k*th element of $\gamma \hat {\pmb \beta }$ and *γ* is eigenvector such that $X^{T} \hat {W}X= \gamma ^{T} \Lambda \gamma $ as *Λ* is a diagonal matrix with eigenvalues of $X^{T} \hat {W}X$ [22, 23]. A study showed that this method works well in reducing MSE [24]. Also, this method has the closed-form, so it saves computation time. Therefore, it was chosen as an estimator for the shrinkage parameter.

### Hypothesis testing about regression coefficients

For testing regression coefficients obtained through maximum likelihood, it is possible to use square root of the main diagonal elements of Fisher information matrix as standard errors of regression coefficients. Then, test statistic is as follows:

$$ t=\frac{\hat{\beta}}{SE({\hat{\beta}})}. $$

This test statistic follows t-distribution. For the penalized maximum likelihood estimators, this test statistic has no longer t-distribution. Some studies have proposed a non-exact t-test for linear ridge regression and logistic ridge regression [25, 26]. For logistic ridge regression, it is as follows:

$$\begin{aligned} Var\left(\hat{\beta}\right)=&Var\left[\left(X^{T}WX+2\lambda I\right)^{-1}X^{T}W \xi\right]\\=&{\left(\frac{\partial^{2} l}{\partial \beta \partial \beta^{T}}\right)}^{-1}I\left(\pmb \beta\right){\left(\frac{\partial^{2} l}{\partial \beta \partial \beta^{T}}\right)}^{-1}\\=& \left(X^{T}WX+2\lambda I\right)^{-1}\left(X^{T}WX\right)\left(X^{T}WX+2\lambda I\right)^{-1} \end{aligned} $$

where $ W=diag\left [\hat {\pi }_{i}\left (1-\hat {\pi }_{i}\right)\right ]$ which *W* is an *n*×*n* matrix, and *ξ* is a vector where the *i*th element equals $\xi _{i}=logit\left [\hat {\pi }_{i}\right ]+ \frac {y_{i}-\hat {\pi }_{i}}{\hat {\pi }_{i}\left (1-\hat {\pi }_{i}\right)}$. Then, the test statistic is:

$$t^{\lambda}=\frac{\hat{\beta_{k}}}{SE\left(\hat{\beta_{k}}\right)}. $$

In this study, the last step of each iteration to estimate the fixed effects uses the Fisher-scoring, so the variance which used in non-exact t-test is:

$$\begin{aligned} Var\left(\hat{\beta}\right)=\left[E\left(\frac{\partial^{2} l^{\lambda}}{\partial \beta \partial \beta^{T}}\right)\right]^{-1}~I(\pmb \beta)~~ \left[E\left(\frac{\partial^{2} l^{\lambda}}{\partial \beta \partial \beta^{T}}\right)\right]^{-1}=\left[{I\left(\pmb \beta\right)}\right]^{-1} \end{aligned} $$

## Numerical study

### Intimate partner violence

In this study, 150 pregnant women referring to health centers in suburbs of Hamadan City (Hamadan Province, Iran) who were under IPV were selected. The study was approved by the ethics committee. This study was conducted in accordance with the Declaration of Helsinki. These women were assigned to control and intervention groups. For the intervention group, 5 public health education sessions were held by a clinical psychologist for 5 weeks. Identifying factors causing IPV and how to manage it, forming support groups of participants, being in contact with the consultant, providing management solutions for these people, increasing communication skills of participants, giving booklets containing conflict management techniques, gift cards, and providing a free counseling session for husbands of these women were a summary of the plans administered in the intervention group.

Before starting the study, a general mental health questionnaire (GHQ) was given to all the participants. At the end of the study, these people again completed this questionnaire. Finally, after data collection, it was attempted to determine effectiveness of the intervention and contribution of various types of violence in psychological aspects of these women. Depression is an important problem in these women. Here, depression was considered as the response variable. Women with depression received a value of 1 and the others received a value of 0. So, the main aim of analysis was assessing effectiveness of the intervention and the effect of types of violence on depression.

At first, types of violence were considered as a matrix, called as *V*. Then, correlation matrix of *V* was obtained, namely *cor*(*V*). As can be seen in *cor*(*V*), there are medium to high correlations between variables. As shown in the *cor*(*V*), there is a warning for the presence of collinearity between these predictors, because most of correlations are above 0.5 [8]. For achieving more assurance about the existence of collinearity, the condition index was computed. This value was equal to 9.8, indicating collinearity between these variables.

$$\begin{aligned} \quad\quad\quad\quad\text{{Financial}\ \ \ {Sexual}\ \ \ {Physical}\ \ \ {Psychological}} \end{aligned} $$

$$\begin{aligned} cor(V)= \left[\begin{array}{ccccccccccccccccccccccccccc} \hspace{6pt} 1 &&&&&&0.47 &&&&0.60 &&&&&&&&&&0.62 \\ &&&&&& 1&&&&0.73 &&&&&&&&&&0.51 \\ &&&&&&&& && 1&&&&&&&&&&0.64\\ &&&&&&&&& && &&&&&&&&&&& 1 \\ \end{array}\hspace{20pt} \right] \begin{array}{c} \text{Financial}\\ \text{Sexual}\\ \text{Physical}\\ \text{Psychological}\\ \end{array} \end{aligned} $$

For modeling, time, intervention, and types of violence were considered. So, the design matrix, X, defined as *X*=[*Intervention*,*Time*,*V*]. Condition number for this matrix was 14.9 which is shows collinearity is a concern. At first, MELM was fitted to these data regardless of collinearity. Then, our proposed model was fitted.

To conducting the global test for the null hypothesis that all of coefficients is simultaneously zero, the likelihood ratio test (LRT) was used. For this data in MELM, the *LRT*=293.91 and *p*−*value*=0.009. This test indicates that all of coefficients is not simultaneously zero. As shown in the first part of Table 1, due to collinearity between predictors, none of predictors is significant at 95% of significance level. Only, psychological violence had a significant effect on depression at 90% of significance level. As can be seen in Table 1, inflation in standard errors is quite obvious. The second part of Table 1 shows the results of our proposed model. As shown in Table 1, standard errors of RMELM are lower than those of the MELM. The standard errors became adjusted and all of variables became significant. The estimated variance of random effects was equal to 1.12 and 1.26 in MELM and RMELM, respectively.

**Table 1:** The impact of types of violence and intervention on depression in IPV women

	*Variable*	$\hat {\beta }$	$SE(\hat {\beta })$	Odds ratio	*P*−*value*
MELM	Group: Control	0.08	0.53	1.08	0.874
	Intervention(Ref)				
	Time	-0.36	0.43	0.69	0.396
	Financial	0.36	0.26	1.43	0.161
	Sexual	0.13	0.24	1.13	0.605
	Physical	0.14	0.13	1.14	0.304
	Psychological	0.09	0.05	1.10	0.079
RMELM	Group: Control	0.44	0.10	1.55	< 0.0001
	Intervention(Ref)				
	Time	-0.86	0.11	0.42	< 0.0001
	Financial	0.87	0.10	2.37	< 0.0001
	Sexual	0.64	0.09	1.90	< 0.0001
	Physical	0.38	0.05	1.46	< 0.0001
	Psychological	0.13	0.02	1.13	< 0.0001

According to the results presented in Table 1, the odds of depression in the control group were 55% higher than the intervention group. Also, the odds of depression were decreased by increasing time so that, odds of depression at time 1 were 2.3 times compared to time 2. Among types of violence, financial violence increased the odds of depression more than other types so that, the odds of depression were increased by 2.37 times in women with the increase in financial violence. After that, sexual violence increased the odds of depression, so that the odds of depression were increased by 90% by increasing sexual violence. As physical violence was increased, the odds of depression were increased by 47%. Finally, as psychological violence was increased, the odds of depression were increased by 17%. All of these factors were significant (*p*−*value*<0.0001).

### Simulation study

For assessing performance of the proposed RMELM, a simulation study was designed and conducted under different settings. Sample size, degree of collinearity between predictors, and correlation between responses were items which considered in the simulation. Here, $\eta _{ij}=\mathbf {x}_{ij}^{T} \pmb {\beta } + \mathbf {z}^{T}_{ij}b_{i}$ was generated with true values for *β*, where, *β*^*T*^=(0.2,0.4,−0.3). Because, there must be collinearity between predictor variables, the correlation between these variables was considered as *ρ*=(0.7, 0.8, 0.9, 0.95). The predictor variables were generated through $x_{ijk}=(1-\rho)^{\frac {1}{2}} ~ a_{ijk} + \rho ^{1/2} ~ a_{ijk}$, where, *i*=1,2,...,*n*,*j*=1,2,*k*=1,2, and *a*_*ijk*_ were generated from standard normal distribution. For investigating the effect of correlation between responses, the intraclass correlation coefficient (ICC) was also considered as *ICC*=(0.2, 0.5, 0.8). RMELM and MELM were compared. MELM was obtained through glmer in lme4 package [27] in R. For assessing performance of these models, relative bias, mean square error (MSE), and empirical power for fixed effects, and variance of random effects were used.

## Discussion and conclusions

In this study, RMELM was introduced for correlated binary responses, and this model was compared with MELM. Table 2 shows the comparative results of MELM and RMELM in terms of MSE and relative bias. For *β*_1_, at n = 30 and ICC = 0.2, MSE for fixed effect estimator in MELM was increased by increasing correlation so that, this value was increased by 2.24 times at correlation level of 0.95 compared to 0.7. At n = 50 compared to smaller sample size, MSE of fixed effect estimator in MELM was relatively smaller and for n = 100, this value was also decreased. With the increase in ICC, MSE for fixed effect estimator in MELM was increased. The increase in MSE at ICC = 0.8 was quite clear compared to ICC = 0.2. The changes in MSE of fixed effect estimator in MELM for *β*_2_ were similar to fixed effect estimator in MELM for *β*_1_. MSE of fixed effect estimator in MELM was small for *β*_3_ compared to *β*_1_ and *β*_2_. Median of relative bias of fixed effect estimator in MELM was equal to 7.5%.

**Table 2:** Comparison of MELM, and RMELM in terms of MSE (relative bias) for fixed effects

			*MELM*			*RMELM*		
*n*	*ICC*	*ρ*	$\hat \beta _{1}$	$\hat \beta _{2}$	$\hat \beta _{3}$	$\hat \beta _{1}$	$\hat \beta _{2}$	$\hat \beta _{3}$
30	0.2	0.7	1.04 (25)	1.03 (17)	0.14 (3)	0.09 (-35)	0.12 (-30)	0.05 (-43)
		0.8	1.38 (5)	1.36 (12)	0.13 (7)	0.10 (-30)	0.14 (-52)	0.05 (-43)
		0.9	2.41 (15)	2.45 (2)	0.12 (0)	0.11 (-25)	0.15 (-55)	0.05 (-43)
		0.95	2.33 (25)	2.46 (-2)	0.12 (0)	0.12 (-25)	0.14 (-57)	0.05 (-43)
	0.5	0.7	1.51 (10)	1.55 (12)	0.18 (0)	0.09 (-45)	0.14 (-60)	0.06 (-53)
		0.8	2.16 (75)	1.95 (-15)	0.18 (-3)	0.10 (-25)	0.15 (-65)	0.06 (-53)
		0.9	3.75 (-50)	3.73 (47)	0.18 (-3)	0.08 (-45)	0.12 (-57)	0.06 (-53)
		0.95	6.93 (-10)	7.38 (27)	0.20 (-3)	0.08 (-30)	0.13 (-62)	0.06 (-53)
	0.8	0.7	3.06 (-30)	3.16 (25)	0.29 (-10)	0.09 (-65)	0.15 (-72)	0.08 (-70)
		0.8	4.34 (35)	4.15 (7)	0.29 (-7)	0.09 (-55)	0.16 (-72)	0.08 (-67)
		0.9	7.56 (5)	7.68 (10)	0.32 (-13)	0.08 (-60)	0.15 (-75)	0.08 (-67)
		0.95	15.12 (25)	15.07 (20)	0.30 (3)	0.06 (-45)	0.14 (-72)	0.07 (-63)
50	0.2	0.7	0.35 (0)	0.39 (7)	0.08 (0)	0.05 (-35)	0.09 (-50)	0.04 (-43)
		0.8	0.60 (-15)	0.61 (12)	0.07 (3)	0.06 (-35)	0.09 (-47)	0.03 (-43)
		0.9	1.16 (-20)	1.09 (15)	0.08 (0)	0.07 (-35)	0.09 (-50)	0.04 (-43)
		0.95	2.21 (10)	2.23 (2)	0.07 (-3)	0.06 (-15)	0.10 (-55)	0.04 (-43)
	0.5	0.7	0.60 (-15)	0.63 (15)	0.10 (-3)	0.07 (-45)	0.10 (-52)	0.05 (-50)
		0.8	1.02 (35)	0.96 (-15)	0.11 (3)	0.06 (-35)	0.12 (-62)	0.05 (-50)
		0.9	1.84 (20)	1.81 (-2)	0.09 (-3)	0.06 (-35)	0.11 (-60)	0.05 (-50)
		0.95	3.7 (20)	3.74 (0)	0.12 (10)	0.05 (-30)	0.10 (-62)	0.04 (-47)
	0.8	0.7	1.75 (15)	1.79 (30)	0.21 (3)	0.07 (-60)	0.13 (-67)	0.06 (-60)
		0.8	2.76 (30)	2.56 (20)	0.18 (-7)	0.07 (-55)	0.14 (-70)	0.06 (-67)
		0.9	5.08 (-5)	5.16 (22)	0.20 (7)	0.06 (-55)	0.13 (-70)	0.06 (-60)
		0.95	9.40 (-15)	9.71 (22)	0.18 (0)	0.06 (-33)	0.13 (-72)	0.06 (-63)
100	0.2	0.7	0.18 (5)	0.18 (-5)	0.03 (0)	0.04 (-30)	0.07 (-47)	0.03 (-37)
		0.8	0.23 (5)	0.24 (-5)	0.03 (-7)	0.04 (-25)	0.07 (-47)	0.03 (-40)
		0.9	0.44 (0)	0.43 (-2)	0.03 (-3)	0.04 (-25)	0.07 (-47)	0.03 (-40)
		0.95	0.88 (15)	0.88 (-15)	0.03 (0)	0.04 (-20)	0.08 (-52)	0.03 (-40)
	0.5	0.7	0.25 (-5)	0.26 (5)	0.04 (-3)	0.05 (-45)	0.07 (-50)	0.03 (-46)
		0.8	0.37 (15)	0.37 (-2)	0.04 (0)	0.05 (-35)	0.08 (-55)	0.03 (-46)
		0.9	0.61 (-10)	0.66 (7)	0.04 (0)	0.04 (-35)	0.09 (-55)	0.03 (-46)
		0.95	1.32 (-25)	1.30 (7)	0.04 (-6)	0.04 (-35)	0.08 (-57)	0.03 (-50)
	0.8	0.7	0.82 (15)	0.90 (15)	0.10 (-6)	0.05 (-55)	0.11 (-65)	0.05 (-66)
		0.8	1.05 (35)	1.15 (2)	0.08 (0)	0.05 (-50)	0.12 (-67)	0.05 (-63)
		0.9	2.05 (35)	2.08 (-7)	0.09 (6)	0.05 (-50)	0.12 (-70)	0.05 (-60)
		0.95	3.95 (-10)	3.88 (25)	0.09 (0)	0.05 (-50)	0.11 (-67)	0.05 (-60)

True values: *β*_1_=0.2,*β*_2_=0.4,*β*_3_=−0.3

Variation of MSE for fixed effect estimators in RMELM was quite different from MELM. At n = 30 and different ICCs, it cannot be said that MSE for fixed effect estimator in RMELM increases by increasing correlation, but these changes have a relatively constant trend. At n = 30 and ICC = 0.2, for estimating *β*_1_, MSE of estimator in MELM was obtained as 11.5, 13.8, 21.9, and 19.4 relative to RMELM. This difference for MSE of the estimator in two models is multiplied as ICC is increased. As the sample size is increased, MSE for fixed effect estimator in RMELMis decreased. Changes in *β*_2_ were similar to *β*_1_. MSE of fixed effect estimator for *β*_3_ in RMELM was less than that of *β*_1_ and *β*_2_. This value was smaller than that of MELM. Median of relative bias was 50% for fixed effect estimator in RMELM.

Table 3 shows empirical power for these estimators. The empirical power of MELM was very small for *β*_1_, and *β*_2_. It was increased about 0.2 by increasing sample size. The empirical power for MELM of *β*_3_ was higher than the other two parameters and its maximum values were obtained in n = 100 and ICC = 0.2. For larger ICCs, this value was decreased. The empirical power of RMELM for *β*_1_ and *β*_2_ was greater than those for MELM. For instance, for *β*_1_, the empirical power in n = 30 and ICC = 0.2 was 16, 19.25, 15, and 18.75 times for RMELM relative to MELM. Table 4 provides estimates regarding variance of random effects for MELM and RMELM. As ICC increased, variance in random effects was increased in both models. With the increase in sample size, the variance was decreased for MELM.

**Table 3:** Comparison of MELM, and RMELM in terms of empirical power

			*MELM*			*RMELM*		
*n*	*ICC*	*ρ*	$\hat \beta _{1}$	$\hat \beta _{2}$	$\hat \beta _{3}$	$\hat \beta _{1}$	$\hat \beta _{2}$	$\hat \beta _{3}$
30	0.2	0.7	0.05	0.08	0.10	0.80	0.82	0.90
		0.8	0.04	0.06	0.09	0.77	0.79	0.88
		0.9	0.05	0.06	0.11	0.75	0.76	0.91
		0.95	0.04	0.04	0.10	0.75	0.73	0.90
	0.5	0.7	0.04	0.06	0.08	0.77	0.80	0.88
		0.8	0.06	0.06	0.09	0.77	0.77	0.88
		0.9	0.04	0.05	0.08	0.71	0.73	0.89
		0.95	0.04	0.04	0.09	0.62	0.66	0.87
	0.8	0.7	0.05	0.04	0.05	0.74	0.74	0.83
		0.8	0.05	0.05	0.06	0.74	0.73	0.84
		0.9	0.04	0.04	0.06	0.67	0.70	0.85
		0.95	0.05	0.05	0.06	0.56	0.57	0.84
50	0.2	0.7	0.05	0.08	0.18	0.88	0.89	0.95
		0.8	0.06	0.08	0.19	0.85	0.89	0.96
		0.9	0.06	0.09	0.21	0.84	0.86	0.94
		0.95	0.06	0.07	0.18	0.81	0.80	0.95
	0.5	0.7	0.06	0.09	0.15	0.86	0.86	0.91
		0.8	0.08	0.07	0.14	0.84	0.87	0.93
		0.9	0.06	0.08	0.17	0.83	0.81	0.93
		0.95	0.05	0.05	0.18	0.75	0.76	0.94
	0.8	0.7	0.06	0.07	0.10	0.85	0.85	0.90
		0.8	0.06	0.06	0.08	0.80	0.81	0.88
		0.9	0.06	0.07	0.10	0.77	0.77	0.90
		0.95	0.07	0.07	0.09	0.72	0.74	0.90
100	0.2	0.7	0.11	0.20	0.46	0.88	0.90	0.98
		0.8	0.09	0.15	0.42	0.89	0.91	0.98
		0.9	0.07	0.11	0.43	0.87	0.90	0.97
		0.95	0.08	0.09	0.43	0.84	0.88	0.98
	0.5	0.7	0.10	0.16	0.34	0.86	0.88	0.94
		0.8	0.10	0.13	0.36	0.87	0.88	0.96
		0.9	0.08	0.10	0.37	0.82	0.87	0.95
		0.95	0.07	0.08	0.32	0.78	0.81	0.95
	0.8	0.7	0.10	0.12	0.20	0.82	0.84	0.88
		0.8	0.10	0.10	0.20	0.80	0.81	0.91
		0.9	0.09	0.10	0.23	0.76	0.77	0.91
		0.95	0.09	0.11	0.22	0.73	0.74	0.92

**Table 4:** Comparison of MELM and RMELM in terms of variance component

*n*	*ICC*	*ρ*	MELM	RMELM
30	0.2	0.7	35.51	16.23
		0.8	55.82	16.39
		0.9	31.61	16.39
		0.95	37.85	16.39
	0.5	0.7	90.42	41.83
		0.8	82.36	41.89
		0.9	69.25	42.28
		0.95	75.14	42.15
	0.8	0.7	203.67	264.10
		0.8	170.74	264.49
		0.9	186.09	264.64
		0.95	169.43	262.87
50	0.2	0.7	10.81	16.44
		0.8	17.38	16.54
		0.9	13.68	16.49
		0.95	15.07	16.52
	0.5	0.7	26.21	42.02
		0.8	37.03	42.20
		0.9	38.22	42.19
		0.95	46.13	42.21
	0.8	0.7	137.90	263.55
		0.8	149.56	261.40
		0.9	148.32	261.48
		0.95	120.76	263.8
100	0.2	0.7	5.04	16.15
		0.8	5.28	16.41
		0.9	4.70	16.39
		0.95	5.07	16.45
	0.5	0.7	15.11	41.08
		0.8	12.90	41.65
		0.9	13.54	41.54
		0.95	11.38	41.77
	0.8	0.7	83.39	256.69
		0.8	78.19	256.61
		0.9	67.40	257.31
		0.95	73.20	259.98

In this study, two methods were used to investigate the effect of different types of violence on depression in pregnant women under IPV. Due to collinearity between types of violence, for MELM, none of the predictor variables was significant at 95% of significance level and only one predictor variable was significant at 90% of significance level. Using the new method, the effect of all types of violence (financial, sexual, physical, and psychological) on depression was significant. These findings illustrate how collinearity influences the results of longitudinal studies with binary responses. The results obtained by the new estimator were consistent with the other previous studies in this area. It has been demonstrated that financial violence influenced depression in Brazilian pregnant women [28]. Physical violence has been also shown to affect the depressed married women in Korea [29]. Results of a study conducted in Tanzania revealed that emotional, physical, and sexual violence affected women’s depression [30].

The results of the simulation study showed that the new model has a lower MSE for fixed effects than the MELM. The new model also increased the empirical power well. Also, in numerical study, inflation in variance of fixed-effects in MELM was shown in the MELM, and a better estimation was made using RMELM.

## Data Availability

The written R codes for the current study are available from the corresponding author on reasonable request. Data sharing is not applicable to this article as the ethical concerns nature of IPV data.

## Abbreviations

MSE: Mean square error
MLE: Maximum likelihood estimator
MELM: Mixed effects logistic model
RMELM: Ridge mixed effects logistic model
IPV: Intimate partner violence
ICC: Intraclass correlation
EM: Expectation maximization
LRT: Likelihood ratio test
